# Is ART suspension never a good idea? - Cons

**DOI:** 10.1186/1471-2334-14-S2-S13

**Published:** 2014-05-23

**Authors:** Guido Poli

**Affiliations:** 1 Vita-Salute San Raffaele University, Milano, 20132, Italy

## 

In the mid ‘90s tremendous progress has marked the pharmacological approach to HIV-1 disease, resulting in the almost complete reversal of the “death sentence” that was associated with the diagnosis of HIV infection and AIDS. However, the terrific advantages of combination antiretroviral therapy (cART) are not available homogeneously on a global scale, with the number of individuals becoming infected still outscoring that of infected people starting treatment. Even restricting the analysis to those countries, including Europe, where cART is largely available, there are several considerations supporting the concept that therapy suspension could still be considered in specific settings, and not only for facing the management of accumulation of multidrug resistant viral species or therapy-related side effects. In fact, some of the most novel and exciting novelties in the field of HIV research have arisen from examples of either casual or programmed therapy suspension. In the case of the so-called “Mississippi Baby”, a newborn (from a mother with undiagnosed AIDS until the time of delivery) was treated with cART shortly after delivery; nonetheless, she became unequivocally infected, but seroreverted after a few weeks. Later, cART was suspended for unknown reasons, surprisingly resulting in the lack of virus rebound and HIV detectability by multiple testing, leading to the hypothesis that she might represent the second case of “cure” after Tymothy Ray Brown, the “Berlin patient”. In a second example, adult French patients who started their antiretroviral regimens very early after primary HIV infection, underwent therapy suspension after a few years resulting in an unexpected high frequency of spontaneous control of HIV-1 viremia and disease progression (Post-therapy controllers, or VISCONTI cohort). The likelihood that they might represent conventional spontaneous controllers of disease progression, such as LTNP or Elite controllers, is minimal in that their genetic profile is enriched in the BW35 MHC allele that is counterselected in these categories. In summary, without these and other example of cART suspension our current understanding of the potential opportunities to tilt the balance in favor of the host rather than of the virus would be more limited and perhaps would prevent future strategic approaches to achieve long-term control of HIV infection with minimal side effects related to cART.

